# Black’s Micro-Organisms

**Published:** 1884-09

**Authors:** 


### BLACK'S MICRO-ORGANISMS.

Just as the last form is ready for the press, we have received the above work. It is too late for review in this number, but it shall have due attention in the future.

We wonder what proportion of the profession will purchase and read this work from an acknowledged authority. Some time since, Dr. T. H. Chandler translated from the French two books of importance, and he has informed us that the most of the editions still moulder upon the shelves of the publisher. And yet, in grandiloquent terms, we have heard the claim boastingly put forth that dentistry, the latest born of fair Science, was the farthest advanced in technical knowledge of all her children. We hope the sale of this book may prove so. But there are too many of us who delight in scientific platitudes, who revel in high sounding, technical phrases, to which no definite meaning is attached, but who, when a calm consideration of some segregated, technically scientific question is presented, take refuge in the denunciation of text-books, and a sweeping condemnation of theoretical speculation. For the credit of the profession we hope that this book will be generally purchased and intelligently studied.

